# Can chatbots help to motivate smoking cessation? A study on the effectiveness of motivational interviewing on engagement and therapeutic alliance

**DOI:** 10.1186/s12889-022-13115-x

**Published:** 2022-04-12

**Authors:** Linwei He, Erkan Basar, Reinout W. Wiers, Marjolijn L. Antheunis, Emiel Krahmer

**Affiliations:** 1 Department of Communication and Cognition, Tilburg School of Humanities and Digital Sciences, Tilburg University, Tilburg, the Netherlands; 2 Behavioural Science Institute, Radboud University Nijmegen, Nijmegen, the Netherlands; 3 Addiction Development and Psychopathology (ADAPT)-Lab, Department of Psychology, and Centre for Urban Mental Health, University of Amsterdam, Amsterdam, the Netherlands

**Keywords:** Chatbot, Smoking Cessation, Motivational Interviewing, Engagement, Therapeutic Alliance, Empathy, Motivation to Quit

## Abstract

**Background:**

Cigarette smoking poses a major threat to public health. While cessation support provided by healthcare professionals is effective, its use remains low. Chatbots have the potential to serve as a useful addition. The objective of this study is to explore the possibility of using a motivational interviewing style chatbot to enhance engagement, therapeutic alliance, and perceived empathy in the context of smoking cessation.

**Methods:**

A preregistered web-based experiment was conducted in which smokers (*n* = 153) were randomly assigned to either the motivational interviewing (MI)-style chatbot condition (*n* = 78) or the neutral chatbot condition (*n* = 75) and interacted with the chatbot in two sessions. In the assessment session, typical intake questions in smoking cessation interventions were administered by the chatbot, such as smoking history, nicotine dependence level, and intention to quit. In the feedback session, the chatbot provided personalized normative feedback and discussed with participants potential reasons to quit. Engagement with the chatbot, therapeutic alliance, and perceived empathy were the primary outcomes and were assessed after both sessions. Secondary outcomes were motivation to quit and perceived communication competence and were assessed after the two sessions.

**Results:**

No significant effects of the experimental manipulation (MI-style or neutral chatbot) were found on engagement, therapeutic alliance, or perceived empathy. A significant increase in therapeutic alliance over two sessions emerged in both conditions, with participants reporting significantly increased motivation to quit. The chatbot was perceived as highly competent, and communication competence was positively associated with engagement, therapeutic alliance, and perceived empathy.

**Conclusion:**

The results of this preregistered study suggest that talking with a chatbot about smoking cessation can help to motivate smokers to quit and that the effect of conversation has the potential to build up over time. We did not find support for an extra motivating effect of the MI-style chatbot, for which we discuss possible reasons. These findings highlight the promise of using chatbots to motivate smoking cessation. Implications for future research are discussed.

## Background

Cigarette smoking poses a major threat to public health, contributing to more than 6 million preventable deaths per year worldwide [1]. Research has demonstrated that aided quit attempts (e.g., with pharmacological and/or behavioral counseling support) are more likely to succeed than unaided quit attempts [2, 3]. While support provided by healthcare professionals is effective, its use remains low. Developing innovative tools that can complement traditional cessation support is, therefore, a research priority. Conversational agents, or chatbots, could be a useful tool for smoking cessation interventions because they are always accessible, can engage users in a human-like conversation, and can provide personalized content to multiple users simultaneously. Additionally, chatbots may appeal to certain hard-to-reach groups such as young people, who often express low motivation to quit smoking and high health technology acceptance [4].

Despite their promise, the current generation of chatbots has yet to fulfill their potential and the effectiveness of chatbot interventions is inconclusive. One crucial factor in effective chatbot interventions is engagement, which has proved to be a significant predictor of improved outcomes for health behavior change in general and for smoking cessation in particular [5–8]. However, current chatbots interventions often suffer from low engagement and high attrition rates[9, 10], and the long-term effect is, therefore, uncertain. Building therapeutic alliance is another challenge for interventions delivered by chatbots. A positive alliance is a robust predictor of addiction treatment outcomes [7, 11] and is especially important for long-term behavior change such as smoking cessation which requires sustained effort. However, in chatbot interventions, it is a crucial but as yet unaddressed question whether therapeutic alliance can develop when the interaction is computerized and automatized without direct involvement of a human therapist [12, 13]. Next to engagement and therapeutic alliance, the importance of empathy is universally acknowledged in various behavior domains including smoking cessation [14], and training in counselors’ empathy has been a research priority [15]. However, it remains a major challenge for a chatbot to be perceived as empathic given its robotic nature [16]. In light of these challenges, the potential of using chatbots in smoking cessation intervention is yet to be fulfilled and it raises the question what the optimal role of chatbots in assisting smoking cessation intervention is. The first aim of this study is, therefore, to explore under which circumstances chatbots can be useful in motivating smoking cessation.

Motivational Interviewing (MI) has the potential to overcome the above-mentioned challenges that hinder the effectiveness of chatbot smoking cessation interventions. MI is a client-centered, directive, yet non-confrontational counseling approach for enhancing motivation to change [17]. Counselors employ the principles of expressing empathy, avoiding arguing, managing resistance without confrontation, and supporting the individual’s self-efficacy; using counseling techniques such as asking open-ended questions, reflective listening, affirming, and summarizing [17, 18]. MI is a versatile approach that can be used as an additive to other interventions, as a prelude to another treatment where it serves as a preparatory role, and as a stand-alone intervention. Several meta-analyses concluded that the effect of basic MI is most pronounced when it works as a pre-treatment prelude [19, 20]. These observations suggest the advantages of employing MI as early as possible in an intervention, for example, into the intake sessions that typically precede participants’ assignment to treatment [21]. The first intake interview in a smoking cessation intervention typically involves an assessment of the client’s smoking behavior (e.g., smoking history and habit) and a brief discussion about the wishes and concerns of the client (e.g., potential reasons to quit) [22, 23]. However, to the best of our knowledge, only one study investigated the effectiveness of integrating MI into a pre-treatment intake session in a clinical program [21], and no research has explored the effect of pre-treatment MI in a chatbot setting, while initial engagement and therapeutic relationship building is important in order to maintain long-term human-chatbot interactions. Therefore, this study adopts the context of the first intake session, aiming to examine the early effects of MI in building a positive impression during the initial chatbot contact.

### Early motivational interviewing increases engagement, therapeutic alliance, and perceived empathy

MI can be an effective approach to building a positive first impression, increasing early engagement, and therapeutic alliance. The central principles of MI include expressing empathy, rolling with resistance, eliciting discrepancy, and supporting self-efficacy [24]. Such principles are keenly matched to what is needed to build early engagement and a therapeutic relationship – such as acceptance, understanding, and compassion [25]. Congruent with the theoretical bases, empirical evidence for MI to increase engagement and therapeutic alliance has been universally observed in various behavior domains including smoking cessation [21, 26]. Moreover, the principles of MI place a strong focus on expressing empathy, which is manifested by reflective listening. Through skillful and deliberate reflection, counselors convey a sense of being present, understanding the clients’ words, feelings, and underlying meaning. The notion that MI is effective in increasing empathy has also been supported in the empirical literature [27, 28].

While the majority of MI studies were conducted in clinical settings and were delivered in face-to-face sessions [29], there is a significant amount of evidence that people should also have positive responses to automated MI delivered by chatbots. Following the CASA (Computers Are Social Actors) framework, a series of studies has demonstrated that people respond in social ways to computers as if they would respond to humans [30]. Of particular relevance to this study, Schulman and colleagues demonstrated that MI spirits expressed by a software agent can be positively evaluated in terms of both MI fidelity and user satisfaction [31]. Following the notion that the effectiveness of MI can be expected in a chatbot setting, we hypothesize that there is a positive effect of chatbot-delivered MI on engagement, therapeutic alliance, and perceived empathy.

### Multi-session effect of motivational interviewing

There is a great variation of MI intensity, which refers to the exposure to MI and can be operationalized by the number of sessions. A recent review on MI interventions for smoking cessation [29] reported that the number of sessions (from 1 to 12 sessions) varied considerably across studies and that the intensity of MI has a positive impact on the cessation rate. Following this, one can expect the effect of MI to increase over time in a multi-session setting. Research in MI provided theoretical support for this notion. A number of possible mechanisms have been proposed to explain the efficacy of MI [32], such that more frequent usage of MI-consistent skills and expressions of MI spirits are associated with better treatment outcomes [24, 28, 33, 34]. These causal processes suggest that in a multi-session interaction, as the expression of MI-consistent skills and spirits is accumulating, more positive outcomes can be expected over time. This assumption is confirmed by recent empirical studies [35, 36]. Moreover, research on human–robot interaction suggests that people’s perception of the agents can be formed and changed in a short time [37]. Therefore, in our two-session study, we hypothesize that the levels of engagement, therapeutic alliance, and perceived empathy increase after the subsequent session in the MI condition. Since no MI element is included in the neutral condition, the increase is not expected in the neutral condition.

### Effect of motivational interviewing on motivation to quit

MI has been proven to be successful in smoking cessation programs [38, 39]. However, the efficacy of automated MI delivered by technology is inconclusive [40], suggesting that the robustness of beneficial MI effects may not translate to automated settings. The majority of studies evaluating automated MI have examined the efficacy of MI as an addition to other treatment such as CBT (cognitive behavioral therapy) [40–42] or as a stand-alone intervention [43–45], and not as in pre-treatment intake sessions. In one study [21] that did integrate MI into the intake session, the effects were measured after active treatment, while the immediate effect of pre-treatment MI remained unstudied. To address the gap, this study explores whether the initial contact with an MI chatbot can have an impact on motivation to quit.

### The role of communication competence

Another important factor for successful counseling is the provider’s communication competence, which is characterized by the provider’s communicative knowledge and skills, such as listening and speaking (verbal and non-verbal) skills and interaction management [46]. Healthcare provider’s communication competence is proved to be a positive predictor of treatment outcomes such as patient cooperation and improved health behaviors [46, 47]. While the importance of communication competence has found initial support in human–human communication, it remains unaddressed whether the current generation of chatbots encompasses such quality and whether there is a relationship between such quality and chatbot intervention outcomes. In the healthcare domain, chatbots’ ability to understand users and communicate health-related information accurately and correctly is a central factor determining the effectiveness of the chatbot [48, 49]. Specifically in MI research, the fidelity of automated MI is of great interest to researchers [40], which raises the question of whether a chatbot can reflect correctly and thus give a feeling of being understood, as a human counselor can. To address this question, this study set out to examine the relationship between people’s perception of the chatbot’s communication competence and the outcomes.

To recapitulate, our main hypotheses and research questions are as follows:

- **H1:** An MI-style chatbot conversation results in more engagement (H1a), stronger therapeutic alliance (H1b), and more perceived empathy (H1c), compared to a neutral-style chatbot conversation.
- **H2:** The levels of engagement (H2a), therapeutic alliance (H2b), and perceived empathy (H2c) increase after the subsequent session in the MI condition, but not in the neutral condition.
- **RQ1**: Does the MI chatbot conversation have an impact on motivation to quit?^1^
- **RQ2**: Is there an association between the perception of the chatbot’s communication competence and engagement (RQ2a), therapeutic alliance (RQ2b), and perceived empathy (RQ2c)?

This study presents a proof-of-concept experiment to explore the possibility of using a chatbot in smoking cessation, using the motivational interviewing approach. It adds to the literature on automated MI and digital smoking cessation interventions in the following ways. First, this is one of the first studies evaluating the effectiveness of integrating MI into the initial chatbot contact (i.e., in an intake and discussion session), aiming to build positive first impression as early as possible, as a first step towards long-term support. Furthermore, this study responds to the question of whether a chatbot encompasses sufficient communication competence and whether this has an impact on users’ perception of the chatbot. There has been great interest in chatbots’ ability to deliver automated MI and the fidelity of such, while the evidence remains scarce at present [40].

## Methods

### Overview of the study design

We employed a between-subjects repeated measures design. Participants were randomly assigned to either the MI condition or the neutral condition and interacted with a chatbot in two sessions. Outcome variables were assessed both between and after the two interactions. The two sessions were designed to simulate a typical intake interview in smoking cessation interventions [22, 23]. In the assessment session, typical intake questions in smoking cessation interventions (e.g., smoking history, nicotine dependence level, and intention to quit) [22, 50, 51] were asked by the chatbot. After approximately 5 min, which resembles the waiting room setting, the feedback session took place, in which the chatbot provided personalized normative feedback and discussed with participants potential reasons to quit.

The experiment was approved by the Research Ethics and Data Management Committee of the Tilburg School of Humanities and Digital Sciences (Identification code: REDC 2021.18) and was conducted in compliance with the ethical and data management regulations of the school. The study design, raw materials, and analysis plan are preregistered at Open Science Forum and can be accessed via https://osf.io/e9bvp/.

### Participants and procedure

Power calculations were conducted using the program G*Power 3.1 [52]. Previous meta-analyses have indicated small to medium effect sizes for MI [19]. An a-priori statistical power analysis with repeated measures ANOVA as the statistical test suggested that a minimum sample size of 150 was adequate to detect small to medium effects and interaction effects (effect size *f* = 0.2, power = 0.8). To be eligible to take part in this study, participants had to be at least 18 years old, had to be able to read and write in English, and to have smoked at least one cigarette during the week before participation.

**Fig. 1:**
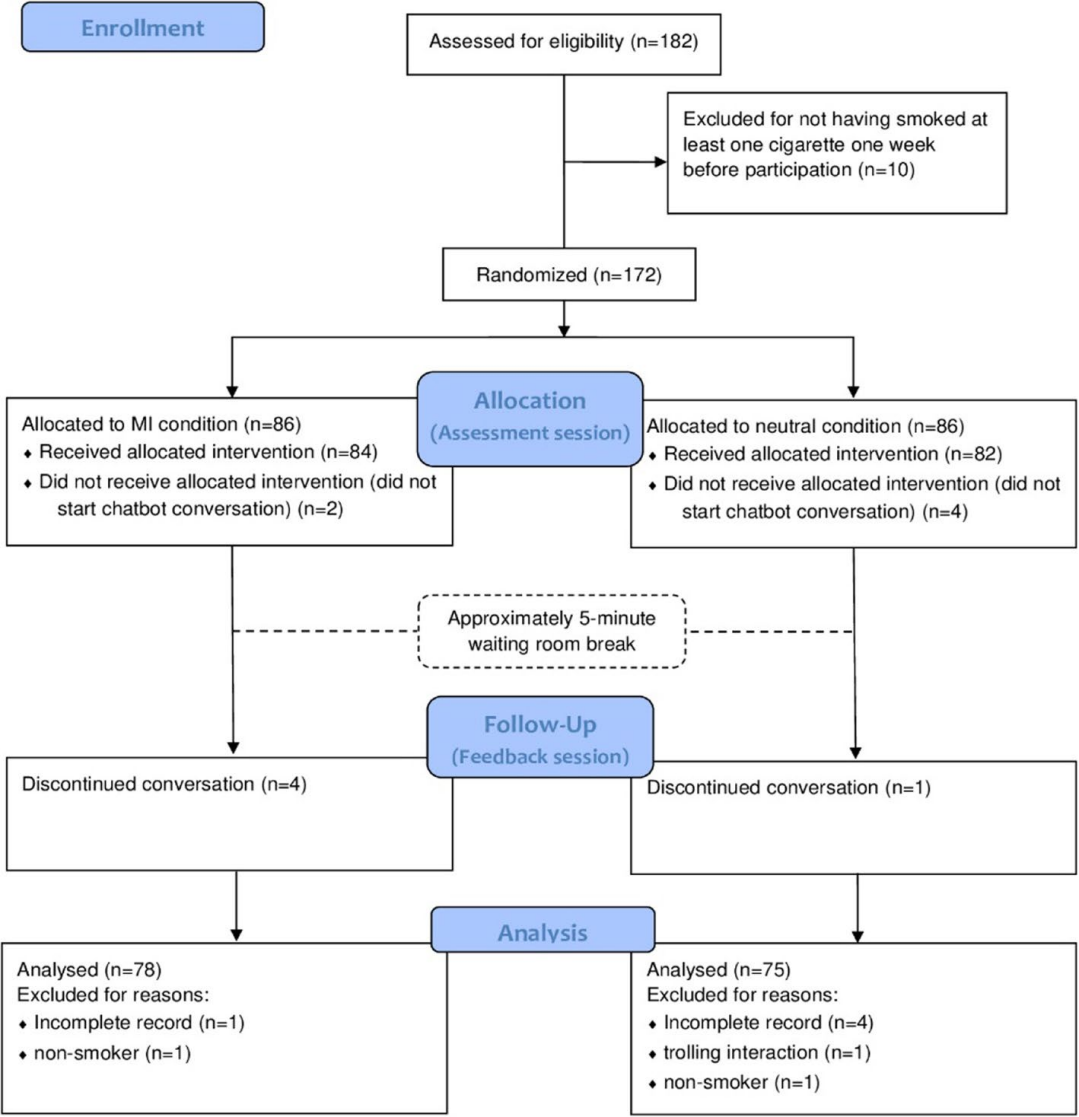
Consort Flow Diagram

Figure 1 depicts the procedure of this experiment. After reading the information letter and agreeing to the informed consent, all participants completed the pre-test questionnaire, which assessed demographics and baseline motivation to quit smoking. Upon starting the experiment, participants were randomly assigned to either an MI or a neutral assessment session, after which they filled out a questionnaire measuring the outcomes (i.e., engagement, therapeutic alliance, perceived empathy, and communication competence). In the feedback session, participants interacted with the chatbot concerning their smoking behavior and potential reasons to quit. After this, relevant variables were assessed in the post-test questionnaire. Upon completion, all participants were debriefed.

### The MI chatbot condition

The practice of MI involves the expression of several core relational qualities and communicative skills that are employed throughout the interaction. The spirits are partnership, acceptance, compassion, and evocation. The communicative skills are asking open questions, reflective listening, affirming, summarizing, and asking for permission before providing information [17]. Here we describe how the spirits and skills were translated into the chatbot scripts for the assessment and feedback sessions.

#### The assessment session

The chatbot first introduced itself and the agenda of the session, after which it interviewed the participants about their smoking behavior. After each answer from the participant, the chatbot responded with a reflective statement. For example, if a participant indicated he or she smokes within 5 min after waking up, the chatbot reflected “You feel that cigarettes help you to start your day.” When a participant indicated abilities in controlling smoking, the chatbot responded with an affirming statement (e.g., “That’s great. You don't have problems refraining from smoking”). Finally, when asking about the intention to quit, the chatbot emphasized autonomy (e.g., “That's okay if you are not ready. It's totally up to you”) to participants who indicated no intention and expressed affirmation (e.g., “You've really made a decision, that’s great!”) to positive intention. More examples of the dialogues are provided in Table 1.

**Table 1:** Example of MI dialogues

MI skill/spirit	Example response
Asking open questions	What do you see as some not-so-good things if you continue smoking as you are? (feedback)
Reflective listening	… you have concerns about the consequences of smoking in that situation. That's great. (assessment)
	I see that health is important to you and it is a concern to you that smoking may impact your health and well-being in the long term. (feedback)
Affirming	And that's certainly okay! You're thinking about it, that's already a first step. (assessment)
	Those are some great points that you bring out, and I'm really glad you came up with these ideas!:). (feedback)
Summarizing	So, let me summarize what I understand so far. There are some important things in your life that you want to take care of, for example, your health and your financial responsibilities, and you're seeing how smoking might impact those important goals of yours. (feedback)
Partnership	We're basically going to discuss a few questions to help both of us get a better understanding of your smoking. Does that sound ok to you? (assessment)
Acceptance	And that's certainly your choice, of course:) If you ever find yourself thinking more about this decision in the future, my *door* is always open! (assessment)
Compassion	It would be a hard time for you without it, I can imagine. (assessment)
Evocation	What, according to you, would be some good things about not smoking? (feedback)

#### The feedback session

The chatbot first provided personalized normative feedback (i.e., the percentage of smokers in their age group) after asking for permission. Then it elicited participants’ reasons to quit by asking open questions (e.g., “What, according to you, would be some good things about not smoking?”) and reflectively listened to the answers (e.g., “So You care about people close to you and you don't want smoking to influence your relationships with them”). When participants did not provide their own reasons (e.g., “I don’t know”), the chatbot provided frequently mentioned reasons [53–55] and let the participants choose the most relevant one (e.g., “I do have some ideas why people might decide not to smoke, but what really matters is what is important to you. Just to help us brainstorm a bit, do any of the following things apply to you?”). At the end of the conversation, the chatbot summarized their previous conversation, thanked the participants, and ended the session.

### The neutral chatbot condition

**Fig. 2:**
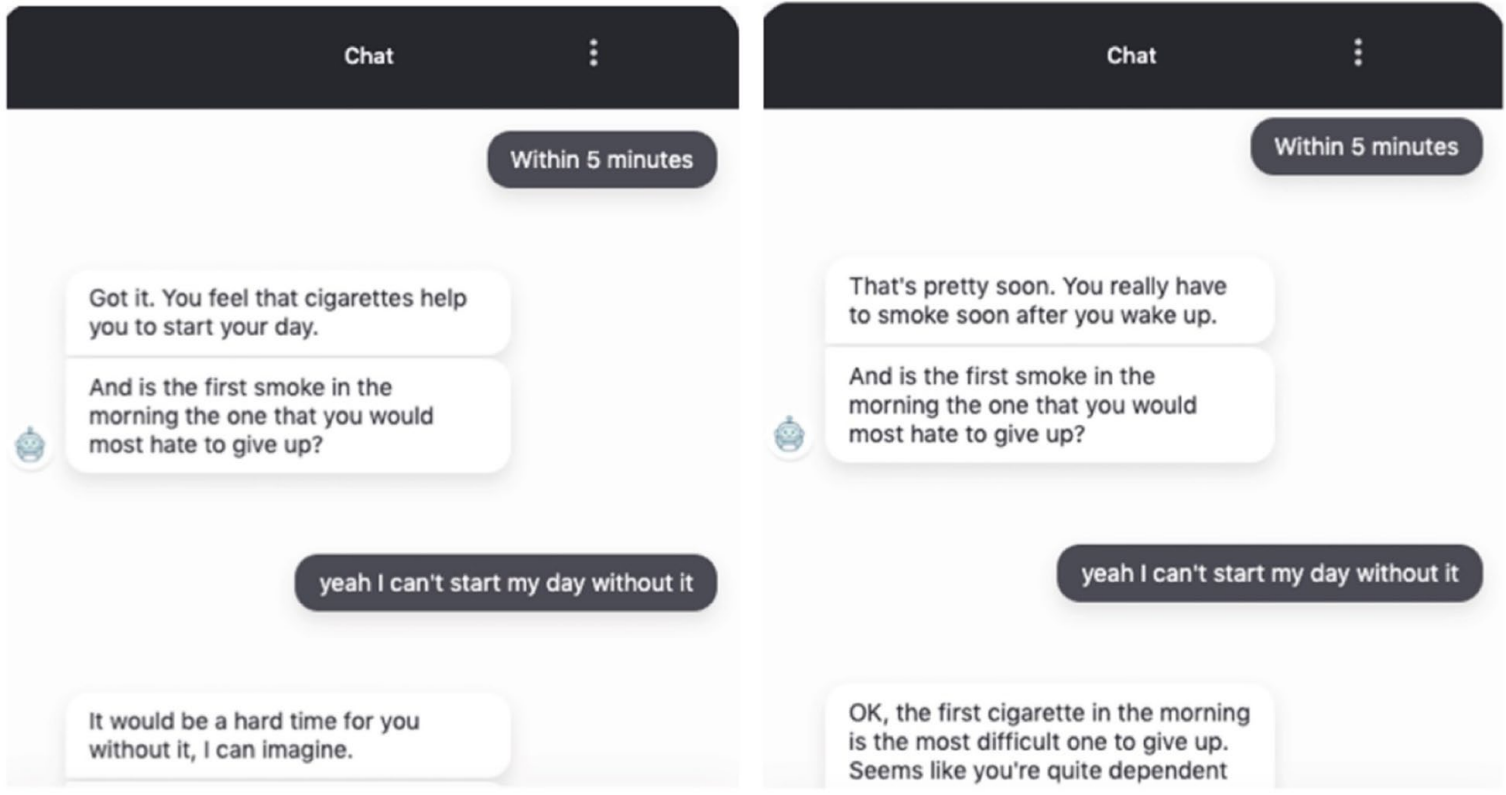
Example conversation assessment session, MI vs. Neutral

**Fig. 3:**
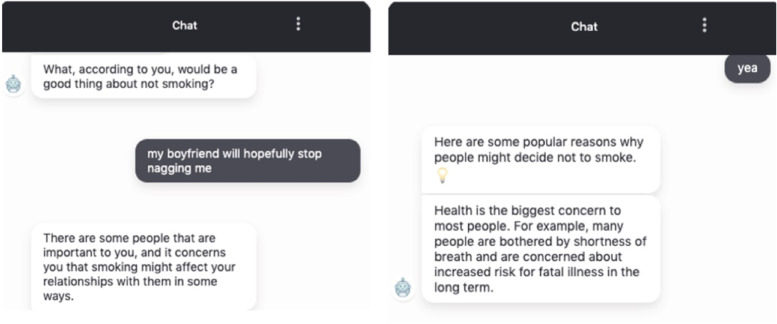
Example conversation feedback session, MI vs. Neutral

The MI spirits and skills were not implemented in the neutral chatbot dialogues. Specifically, when the MI chatbot responded reflectively, the neutral chatbot used conversational fillers (e.g., “Ok, thanks”, “Got it, let’s move on”) and repeated user input (e.g., “you smoke within 5 min after waking up”). When the MI chatbot asked open questions to elicit participants’ reasons to quit, the neutral chatbot provided common reasons to quit. While the MI chatbot always asked for permission before sharing information, the neutral chatbot provided information directly. Except for the aforementioned manipulations, the content of the dialogues (e.g., questions, the order of questions, the information provided) was identical across the two conditions. See Fig. 2 and Fig. 3 for an example of the final conversation. The full scripts can be accessed via https://osf.io/e9bvp/.

The chatbot named Roby was developed using the flow.ai program, a chatbot-building platform frequently used by companies and researchers. The chatbots can be accessed via https://widget.flow.ai/try/YXc2RXpycnF0fGNCNzFMcHBkRA =  = (MI condition) and https://widget.flow.ai/try/YXpfQ0VBY1VKfGNYaGJqc0JFYw =  = (neutral condition).

### Measures

#### Engagement with the chatbot

Engagement with the chatbot interaction was assessed with 9 items from sub-scales of the short form of the User Engagement Scale [56], a measure of engagement with human–computer interaction that has been used in a variety of digital domains. The aesthetic appeal sub-scale was removed as the present study focuses on the communication process instead of the interface design. Example items from the scales were: “I found the conversation confusing,” “I felt interested in talking with Roby.” An additional question was included, asking about participants’ endorsement for future use. The response categories ranged from 1 (completely disagree) to 5 (completely agree).

#### Therapeutic alliance

Therapeutic alliance was measured using the Working Alliance Inventory-Short Revised [57], a 12-item self-report measure used to assess the relationship between the therapist and the client. This instrument is composed of three subscales: a goal-subscale (e.g., “Roby and I are working towards mutually agreed upon goals''), a task-subscale (e.g., “As a result of these sessions I am clearer as to how I might be able to change”), and a bond-subscale (e.g., “I feel that Roby appreciates me”). The response categories ranged from 1(completely disagree) to 5 (completely agree).

#### Perceived empathy

Perceived empathy was measured with a 3-item scale based on research on interpersonal communication by Rubin and Martin [58]. Participants indicated to what extent they agreed with the following statements: “Roby seems to know how I was feeling,” “Roby seems to understand me,” and “Roby puts itself (or himself or herself) in my shoes.” The response categories ranged from 1 (completely disagree) to 5 (completely agree).

#### Communication competence

Following research by Croes and Antheunis [59] on social chatbots, communication competence was measured with the following items: “Roby communicated properly,” “Roby communicated correctly,” “Roby came across as competent,” and “Roby came across as believable.” The response categories ranged from 1 (completely disagree) to 5 (completely agree).

#### Motivation to quit

Motivation to quit was measured with the Contemplation Ladder, a single-item scale developed by Biener and Abrams [60] to assess readiness to stop smoking. Participants indicated where they identify themselves on an 11-point Likert-type ladder with 10 rungs, anchoring at the bottom with 0 (no thoughts on quitting) and at the top with 10 (taking action to quit).

#### Perception of motivational interviewing

As a manipulation check question, perception of MI was measured with an 8-item scale based on the Client Evaluation of Motivational Interviewing Scale [61]. Example items were: “Roby argued with you to change your behavior” and “Roby helped you feel confident in your ability to change your behavior.” The response categories ranged from 1 (completely disagree) to 5 (completely agree).

### Statistical analysis

An independent samples t-test was conducted to test whether the manipulation of MI style was successful. To check for equal distribution of background variables across conditions, an independent samples t-test and Chi-square tests were conducted. Hypotheses and RQ1 were tested with a series of repeated measures ANOVAs. In each analysis, condition was included as the between-subjects factor and time as the within-subjects factor. The outcome variables were engagement, therapeutic alliance, and perceived empathy, respectively. To answer RQ2 regarding the relationship between perceived communication competence and the outcomes, a series of correlation analyses were conducted for T1 and T2, respectively.

## Results

### Participants characteristics and descriptive outcomes

From April 30 to May 29, 2021, a total of 182 participants were recruited via the participant pools from Tilburg School of Humanities and Digital Sciences and Tilburg School of Social and Behavioral Sciences. They received research credits in exchange for their participation. Informed consent was obtained from all participants and/or their legal guardian(s). Each participant received a unique survey ID, which corresponds to individual conversation records with the chatbot. We screened the conversation records to ensure valid participation. 12 participants were removed for not being smokers, 5 for not having a conversation record with the chatbot, 1 for trolling the chatbot conversation (i.e., the participant sent irrelevant messages in the entire conversation), and 11 for having an incomplete conversation with the chatbot, leaving a final sample of 153 participants.^2^ Among these 153 respondents, 62.1% were female, and the majority (87.6%) were born between 1996 and 2003. Characteristics of participants included in the analyses are summarized in Table 2.

**Table 2:** Characteristics of participants

		MI condition		Neutral condition		Total sample	
Variable ^a^		*n (%) or M (SD)*		*n (%) or M (SD)*		*n (%) or M (SD)*	
Total *N*		78	100%	75	100%	153	100%
Gender	Female	51	65.4%	44	58.7%	95	62.1%
	Male	27	34.6%	31	41.3%	58	37.9%
Year of birth	1996–2003	70	89.7%	64	85.3%	134	87.6%
	1991–1995	5	6.4%	9	12%	14	9.2%
	1981–1990	3	3.9%	1	1.3%	4	2.6%
	Before 1981	0	0	1	1.3%	1	0.6%
Daily cigarette consumption		4.6	4.5	5.7	4.2	5.1	4.4
Years of smoking		3.8	3.2	4.6	3.8	4.2	3.5

*Note.*^a^ Gender and year of birth were measured in the pre-test questionnaire; daily cigarette consumption and years of smoking were interviewed by the chatbot

All measures used demonstrated acceptable reliability, see Table 3 for reliability and descriptive results of the outcome variables.

**Table 3:** Means, standard deviations, and reliability results for outcomes

	baseline		T1			T2		
	Mean	SD	Mean	SD	Cronbach’s *α*	Mean	SD	Cronbach’s *α*
Engagement			3.40	0.72	0.85	3.40	0.77	0.87
Therapeutic Alliance			3.25	0.77	0.91	3.33	0.84	0.93
Perceived Empathy			3.36	1.00	0.85	3.40	1.13	0.91
Communication Competence			4.14	0.66	0.79	4.16	0.72	0.84
Motivation to Quit	6.08	2.91				6.72	2.71	

### Manipulation and randomization check

Of all eligible respondents, 78 (51.0%) were assigned to the MI condition and 75 (49.0%) to the neutral condition. The manipulation check results showed that participants in the MI condition perceived the chatbot as more MI-style (*M* = 3.72, *SD* = 0.56) than participants in the neutral condition (*M* = 3.50, *SD* = 0.60), and even though it is small, this difference was statistically significant, *t* (151) = 2.33, *p* = 0.021. Thus, the manipulation was deemed successful.

The randomization checks indicated that there were no significant differences across conditions in terms of gender and baseline motivation to quit. Participants in the MI condition was on average 1 year younger than participants in the neutral condition. However, considering the small difference, age was not included as a covariate in the following analyses.^3^

### Main analyses

H1 posits that an MI-style chatbot conversation results in more engagement (H1a), therapeutic alliance (H1b), and perceived empathy (H1c), compared to a neutral-style chatbot conversation. For engagement, there was no significant effect of condition *F* (1,151) = 0.67, *p* = 0.414, indicating that participants in the MI condition were not more engaged with the chatbot than participants in the neutral condition. Similarly, for therapeutic alliance, no significant differences were found *F* (1,151) = 0.041, *p* = 0.841. Last, no significant effects of condition emerged for perceived empathy *F* (1,151) = 0.57, *p* = 0.452. Therefore, H1 was not supported. See Table 4 for means and standard deviations for dependent variables for both conditions.

**Table 4:** Means and standard deviations for outcome variables for both MI and Neutral conditions

		T1		T2	
		*M*	*SD*	*M*	*SD*
Engagement	MI	3.46	0.72	3.43	0.72
	Neutral	3.34	0.72	3.37	0.82
Therapeutic alliance	MI	3.25	0.74	3.35	0.81
	Neutral	3.24	0.80	3.30	0.87
Perceived empathy	MI	3.43	1.02	3.46	1.07
	Neutral	3.29	0.98	3.35	1.19

H2 predicts that the levels of engagement (H2a), therapeutic alliance (H2b), and perceived empathy (H2c) increase after the subsequent session in the MI condition, but not in the neutral condition. For engagement, repeated measures ANOVA revealed no significant effect of time *F* (1,151) = 0.00, p = 0.966, or interaction effect between condition and time *F* (1,151) = 0.29, p = 0.590. Therefore, H2a is not supported. For therapeutic alliance, while no significant interaction effect emerged for condition by time *F* (1,151) = 0.25, *p* = 0.620, there was a significant main effect for time *F* (1,151) = 4.81, *p* = 0.030. Participants in both conditions indicated a higher therapeutic alliance with the chatbot after the second session. Hence, H2b was not supported. Last, for perceived empathy, no main effect of time was found *F* (1,151) = 0.79, *p* = 0.377, nor an interaction between condition and time *F* (1,151) = 0.08, *p* = 0.779. Therefore, H2c was also not supported.

**Fig. 4:**
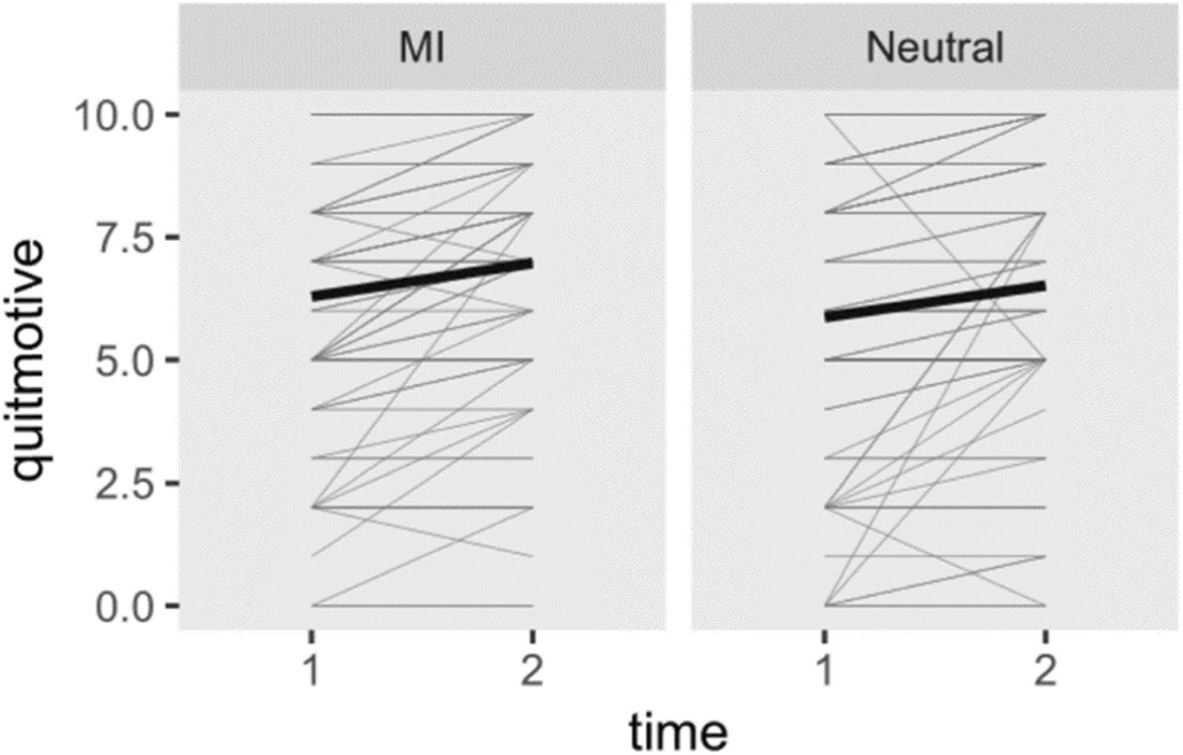
Individual growth in motivation to quit and group means for MI and Neutral conditions

RQ1 concerns the effect of MI-style chatbot conversation on motivation to quit. A significant main effect of time emerged *F* (1,151) = 32.67, *p* < 0.001, indicating that participants in both conditions showed an increase in motivation to quit after the conversation with the chatbots. No significant effects were found for condition *F* (1,151) = 1.01, *p* = 0.317, nor for the interaction between condition and time *F* (1,151) = 0.01, *p* = 0.911. Figure 4 displays the individual change in motivation to quit for both conditions.

Results showed that perceived communication competence was positively correlated with engagement at both T1 *r* (151) = 0.47, *p* < 0.001, and T2 *r* (151) = 0.63, *p* < 0.001. The positive relationship was also observed between perceived communication competence and therapeutic alliance at both time points (T1: *r* (151) = 0.46, *p* < 0.001; T2: *r* (151) = 0.57, *p* < 0.001). Lastly, the same pattern was found for perceived empathy (T1: *r* (151) = 0.50, *p* < 0.001; T2: *r* (151) = 0.52, *p* < 0.001). Hence, there was a positive correlation between perceived communication competence and engagement with the chatbot, therapeutic alliance, and empathy.

## Discussion

### Main findings

This study explored the possibility of using an MI-style chatbot to enhance engagement, therapeutic alliance, and perceived empathy in the context of smoking cessation. Counter to our hypotheses, no significant effect of the experimental manipulation (i.e., MI vs. neutral chatbot) emerged for all the outcomes. Overall, participants who interacted with either the MI-based chatbot or with the neutral chatbot perceived the chatbot as similarly engaging and empathetic, and they built similar levels of therapeutic alliance with their chatbot. There may be several reasons why we did not find positive effects of MI performed by a chatbot. First, the conversations were fairly short and there may have been insufficient time to discover an effect of the MI style. Previous reviews on MI for smoking cessation suggest that there is great variation in the duration of the interventions and that more intensive MI often produces more positive effects than less intensive ones [29]. Among the reviewed studies, similar to this research, several interventions with a duration shorter than 20 min did not find significant effects of MI [62, 63]. This finding is confirmed by results reported by Seal and colleagues [64], indicating that brief MI by telephone had no effect compared to control, but when intensified with higher frequency and length, the positive effect of MI emerged. These findings imply that it might require a longer time for MI to be effective. Notably, both the MI-style chatbot (*M* = 3.72, *SD* = 0.56) and the neutral-style chatbot (*M* = 3.50, *SD* = 0.60) received high ratings in terms of the use of MI skills and spirits. Although we adjusted the neutral dialogues towards the confrontational style, the chatbot was still perceived as motivating, and there might not have been sufficient differences for the MI style to have a manifest effect. Nonetheless, the relatively high motivating ratings of the neutral chatbot can be viewed as a strength of this study, as it implies that using chatbot conversations can mitigate the negative perception of confrontation, even without an explicit MI-style interaction. This is supported by research showing that chatbots can be perceived as less intrusive and less autonomy-threatening, compared to their counterparts such as humans [65]. These findings highlight the potential of chatbots in designing unintrusive persuasion.

This study attempted to explore the multisession effect of MI and hypothesized that the levels of engagement, therapeutic alliance, and perceived empathy to be higher after the second session than after the first session in the MI condition. No increase was found for engagement and perceived empathy, which could be accounted for by the short timeframe of this study. An increase was observed for therapeutic alliance in both conditions. A closer look at sub-concepts of therapeutic alliance revealed that the growth in alliance was found for the task and the goal aspects, but not for attachment bond. One explanation could be that during the second session the chatbots discussed the pros and cons of smoking, and the provided factual information and joint discussion contributed to the increased alliance regarding the tasks and goals, suggesting that people appreciate a chatbot that provides relevant information [66, 67]. This study attempted to feature a multisession scenario and to observe how people’s perception of the chatbot evolved; the results indicated that an increase was found for the task and goal subconcepts of therapeutic alliance but not for the more relational factors (i.e., engagement, therapeutic bond, and empathy), highlighting the need for future research to further examine the process of long-term relationship building.

Results of this study showed that regardless of the conversational style, participants in both conditions reported an increased motivation to quit after talking with the chatbot. This finding supports the literature suggesting that minimal conversation about smoking cessation can affect quitting intentions and behaviors [68, 69]. This answers our question of what role a chatbot can play in the smoking cessation process – merely talking with a chatbot about smoking cessation has the potential to influence people’s thinking about quitting smoking. However, this result should be interpreted with caution considering the demand effect that could have occurred, where participants behave in a way to support the hypotheses. Moreover, a rich body of research has indicated that people tend to underreport unhealthy behavior and overreport intentions to improve and perform actual healthy behaviors [70], which further questions the validity of the self-reported motivation to quit. While our results suggest the potential of chatbot conversation in motivating smokers to quit, future research is recommended to include more objective instruments such as biochemical and behavioral measures to validate the effects of chatbot interventions [71].

In light of the importance of healthcare providers’ communication competence and the lack of research in such quality for chatbots, we set out to explore the relationship between communication competence and the primary outcomes (i.e., engagement, therapeutic alliance, and perceived empathy). Overall, participants perceived the chatbot as highly competent (T1: *M* = 4.14, *SD* = 0.68; T2: *M* = 4.16, *SD* = 0.72). Positive correlations were found between communication competence and all the outcomes, suggesting that a competent chatbot is more likely to produce positive intervention outcomes. It should be noted that we measured communication competence with self-reported questionnaire items, while some studies adopted objective performance measures (e.g., number of chatbot errors) [72]. An examination of conversation records between participants and the chatbot showed that the chatbot did make some mistakes during the experiment (e.g., misunderstanding user input and replying with wrong answers) yet was perceived as highly competent by the participants, indicating there is a mismatch between perception and actual chatbot performance. In order to better understand the role of communication competence, future research could consider including both subjective and objective measures of chatbot performance.

### Strengths and limitations

This is one of the first studies exploring the use of chatbots for increasing smokers’ motivation to quit. One strength of this study results from the preregistration of the design, materials, and raw data, which enables replication and provides training examples for future chatbot development. Moreover, the fact that the conversation took place in two sessions serves as a first step towards the long-term multi-session intervention. The low dropout rate (i.e., 5 out of 165 participants dropped out from the second session) indicates the potential for long-term interactions.

This study shed light on the use of motivational interviewing chatbots, however, there are several limitations that warrant consideration in the interpretation of the findings. First, we compared MI with a neutral-style chatbot, the differences between which might have been too subtle to result in a significant effect. In addition, the development of relational feelings such as engagement and therapeutic alliance might need a longer time than the duration of conversation in the current study. Our finding that therapeutic alliance did increase over the two brief sessions indicates the potential growth, and future studies are, therefore, needed with a longer duration and the possibility for the users to continue the conversation to further examine the relational process. Moreover, despite the growth observed, the break (approximately 5 min) between the two sessions might have not been sufficient to uncover the real multi-session effect, which is often found in studies with higher session frequencies and longer between-session break [35, 36]. Nevertheless, people’s impression on chatbots can evolve in a short time [37], and future research is encouraged to explore this effect with more sessions spread in a longer period of time.

### Implications for future research

The exploratory nature of this study allows us to draw a few implications for future research. First, future designs could consider increasing the contrast between conditions, such as employing a more directive-confrontational style chatbot [73], to better capture the effect of MI. Furthermore, it’s unclear what chatbot features contributed to the increased motivation to quit. Previous research has attempted to disentangle the effects of specific MI techniques and determine the active ingredients of MI [74, 75]. For example, Apodaca et al. [76] found that affirmation was the only technique that promoted change talk and reduced sustain talk. Another finding is that simple reflections were equally effective as complex reflections; this finding is particularly relevant for chatbot-delivered MI, as complex reflections are challenging for the current chatbots. Future research would benefit from identifying techniques that are most effective in a chatbot setting and, therefore, magnify the effectiveness of MI. Moreover, the finding that regardless of condition, participants overall experienced an increase in motivation to quit highlights the promise of using chatbots in health interventions. Previous research has demonstrated that minimal interpersonal communication about health behavior can impact people’s attitude towards and intentions to perform the behavior [69], and our results suggest that human-chatbot communication could have similar effects. Human-chatbot communication and interpersonal communication are similar in many ways. For example, both interlocutors can initiate the conversation and the content of the conversation can vary (e.g., about the user vs. about other smokers/non-smokers), which are factors moderating the effect of the conversation [69, 77]. Future research is suggested to further explore these factors in designing chatbot dialogues.

While chatbots can simulate human-like interaction, they should be a supplementary service rather than a replacement of the human healthcare providers. It’s important to acknowledge that the addition of chatbots has the potential to improve healthcare services notably in relation to anonymity, accessibility, and personalization [78]. Ethical issues such as the balance between anonymity and personalization should be taken into consideration in the development of healthcare chatbots. Given the novelty and the rapid development of such technology, the optimal role of chatbot in assisting cessation services is yet to be determined. Future work should further explore approaches to effectively and safely integrating chatbots into clinical care for smoking cessation.

## Conclusion

The proof-of-concept study set out to explore the possibility of using a chatbot to increase young smokers’ motivation to quit smoking, using the motivational interviewing approach. Overall, we found no significant effects of MI. However, participants in both groups demonstrated an increase in motivation to quit after the conversation, suggesting that interacting with a chatbot about smoking cessation can motivate smokers to quit. An increase in therapeutic alliance emerged after two sessions, indicating the potential for the effect of the conversation to build up over time. These findings highlight the positive outlook of using chatbots to motivate smoking cessation.

## Data Availability

The datasets used during the current study are available from the Dataverse repository at https://doi.org/10.34894/AZ5QJ9.

## Abbreviations

MI: Motivational interviewing
CASA: Computers Are Social Actors
CBT: Cognitive behavioral therapy

## Footnotes

1 Original preregistered RQ (https://osf.io/e9bvp/): Does (RQ1a) and which of (i.e., MI vs. neutral, RQ1b) the conversations with the chatbot have an impact on motivation to quit?

2 According to our preregistration (osf.io/e9bvp), participants who did not complete either of the two entire measurement points would be excluded from the analyses. However, we decided to include having valid conversation records as an additional inclusion criterion to ensure that the observed effects were from the chatbot conversation.

3 We ran the analyses both with and without age as a covariate, and the pattern of results remained the same in terms of significant differences. Here we report results without age as a covariate.
